# An opportunity to improve Acute Ischemic Stroke care in the South Asian region through telestroke services

**DOI:** 10.1016/j.amsu.2021.103115

**Published:** 2021-11-25

**Authors:** Jayant Kumar Yadav, Gaurav Nepal, Yow Ka Shing, Rohit Raman Banerji, Bikram Prasad Gajurel

**Affiliations:** a Department of Internal Medicine, Tribhuvan University Teaching Hospital, Kathmandu, Nepal; b Department of Internal Medicine, National University Hospital, Singapore; c Department of Neurology, Tribhuvan University Teaching Hospital, Kathmandu, Nepal

**Keywords:** Stroke, Ischemic stroke, South Asia, Telestroke, Telemedicine

## Abstract

The South Asian region accounts for more than 40% of the stroke burden of the developing world and is the highest contributor to global stroke mortality. Despite proven treatment guidelines, the limited number of neurologists, the number of dedicated stroke centers and the fact that most of these facilities are urban-centric renders poor access to thrombolysis in this region, especially in the rural areas. Studies have shown that thrombolysis using telestroke services are comparable to face-to-face thrombolysis. Telestroke, conducted through low-cost media such as smartphones or laptops, may form a cost-effective solution to improve access to appropriate stroke care in a resource-limited setting such as that of the South Asian region.

The region of South Asia refers to a geological union of countries bordered by the Himalayan mountain range in the North and the Indian Ocean in the South, comprising Nepal, Bangladesh, Sri Lanka, Bhutan, India, Maldives, and Pakistan (Fig. 1), which share similar ethnocultural and dietary practices. Home to one-fourth of the world's population, this is the most densely populated region in the world and is presently in the midst of a significant epidemiological transition. The burden of non-communicable diseases is on a consistent upward trend owing to rapid urbanization, increasing ubiquity of a sedentary lifestyle, and unhealthy diet choices. Currently, more than 80% of cardiovascular deaths occur in low- and middle-income countries which include the South Asian countries [1]. This is largely driven by the high intake of dietary fat, sugar, and salt, in addition to high tobacco consumption, either by chewing or smoking [2].

**Fig. 1:**
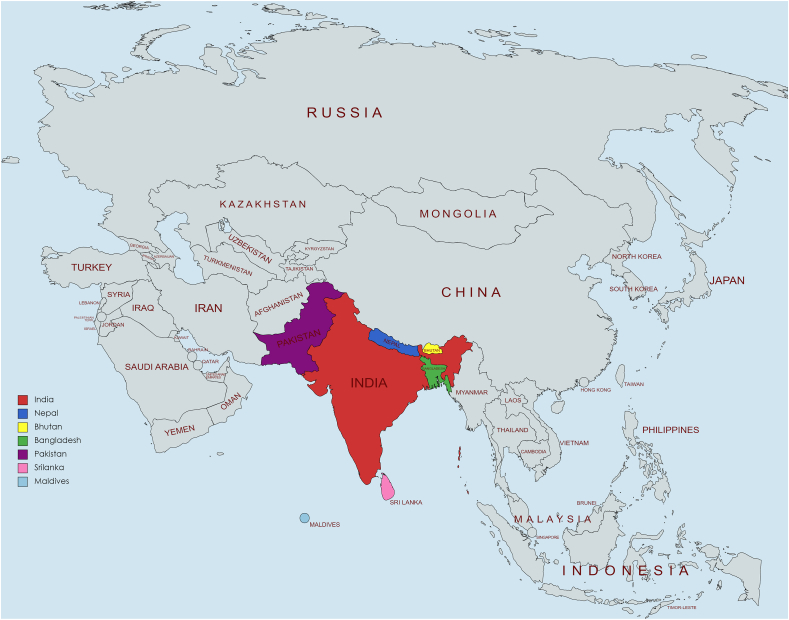
Map of South Asian region.

Traditional South Asian cuisine is known for the significant use of ghee, mustard oil, or coconut oil and frying as a method of cooking. To enhance flavor, there is a trend in reusing the cooking oil in this region, which leads to the production of total polar compounds and polycyclic aromatic hydrocarbons, both of which significantly increase cardiovascular risk. Besides, high heat cooking which is favored in this region promotes neo-formed contaminants (NFCs) such as *trans*-fatty acids (TFAs) and advanced glycation-end products (AGEs). Globalization of fast food establishments has introduced foods low in fiber and rich in energy, saturated fat, and sugar. These have been hypothesized to contribute to the risk of cardiovascular disease in the population.

The South Asian region accounts for more than 40% of the stroke burden in the developing world and is the highest contributor to global stroke mortality [3]. Despite the high stroke burden, South Asia has fewer than 3000 neurologists and has a limited number of dedicated stroke centers (Table 1). The majority of these resources are urban-centric, further limiting the accessibility of neurological services to the larger demographic group. Acute Ischemic Stroke (AIS) accounts for up to 85% of all stroke cases and is highly treatable if the patient presents within the window time for definitive therapeutic intervention such as intravenous thrombolysis or mechanical thrombectomy [4]. Despite the established guidelines, access to thrombolysis remains relatively low [[5], [6], [7], [8]] [[5], [6], [7], [8]] [[5], [6], [7], [8]], especially so in the rural areas. This implies that patients who have an AIS in an urban setting have a much higher chance of obtaining timely therapy compared to their rural counterparts, who often succumb to the ailment and are left with lifelong disability and dependence.

**Table 1:** Demography and available facility for stroke treatment in South Asian Countries.

Countries	The total population of the country	Number of Neurologists [Personal communication]	Number of dedicated stroke centers [Personal communication]
Bangladesh	160 million	150	20 centers capable of IV thrombolysis (IVT) and 1 capable of mechanical thrombectomy (MT)
Nepal	30 million	18	10 centers capable of IVT and 3 capable of MT
India	1.4 billion	2500	100 centers capable of IVT and 55 centers capable of MT
Pakistan	210 million	150	10 centers capable of IVT and 1 capable of MT
Bhutan	0.75 million	None	None
Sri Lanka	21 million	45	10 centers capable of IVT and 4 capable of MT
Maldives	0.51 million	5	3 centers capable of IVT

Telestroke, the use of telemedicine in neurology, is a potential solution to the issue of acute shortage and uneven distribution of neurologists. Telestroke uses state-of-art synchronous audio-video technology to provide virtual care for patients suffering from AIS in remote locations. Telestroke often utilizes a "hub and spoke” model for delivering AIS care. In this model, the tertiary level hospitals or the stroke centers (with stroke specialists/neurologists) function as the hub, while the peripheral centers (without specialists/neurologists) function as the spokes. This way, any trained physician located in a peripheral center can conduct a rapid clinical assessment and conduct initial investigations. Thereafter, the physician can potentially initiate thrombolysis therapy, and if necessary, refer the patient to a tertiary care facility for further evaluation and potential neurovascular intervention. This is also known as the “drip and ship” model, where the intravenous alteplase is given within the critical therapeutic window, and the subsequent continuation of care is handed over to a specialized stroke center or unit. This provision can be used across cities and even across international borders, which can be especially useful for countries like Bhutan where there are no full-time neurologists or stroke treatment centers. This model has already been adopted by the Lao People's Democratic Republic, and they have benefited greatly from teleconsultation from Thailand [9].

Telestroke provides two major advantages: firstly, it helps address the shortages of stroke specialists and centers, and secondly, it helps to narrow the therapeutic time window for treatment with thrombolysis by reducing door-to-needle time [10]. This is important as thrombolysis can be done only for patients with ischemic stroke who present to the hospital within 4.5 h of symptom onset. In a meta-analysis conducted by Baratloo et al., telestroke significantly reduced both onset-to-door and hospital-stay durations in AIS patients without increasing the risk of mortality or symptomatic intracranial hemorrhage [11].

An actual launch of telestroke services came into fruition in the Indian state of Himachal Pradesh in 2014. All government hospitals with CT scans in the region were included in the program. Workshops were conducted to train and enable medical officers in identifying stroke imaging. Intravenous alteplase was made available at all peripheral centers involved and was provided free of charge. Additionally, neurologists were rostered round the clock and were phone-consulted whenever a patient suffering from AIS came in within the window period. The initial results showed a lot of promise. In the first year of the program, 26 patients received thrombolysis and only two patients developed non-fatal intracranial hemorrhage with no acute in-hospital mortality [12]. This is evidence that this is a potentially scalable low-cost and effective solution.

There are admittedly set-up logistics and costs required for establishing a telestroke network, which may be a prohibitive barrier for resource-limited countries such as those in South Asia. However, studies have shown that in the long run, the potential savings and improvement in healthcare indicators, outweighed the cost related disadvantage. This largely stems from the benefit of stroke treatment in preventing lifelong disability and dependence [13]. Studies have found that economic alternatives such as mobile smartphones and laptops to be equally accurate and reliable compared to medical monitors in interpreting the cranial CT images of the patients who had symptoms of AIS. Smartphone teleconferencing was also found to be reliable in the administration of the NIHSS score. Besides, this telehealth facility can be used for other important measures such as patient education, cardiovascular risk modification, post-stroke rehabilitation and assistance in the treatment of other neurological conditions [[13], [14], [15], [16]].

Despite the benefits of a telestroke service, infrastructure, manpower, financial and medico-legal issues are important considerations in the installment of such a service. Firstly, there is concern of effective communication between the patients and the care provider through a telecommunication medium, even more so across cities or countries as internet connection and language barriers may pose a crucial impediment to accurate and timely medical advice and treatment. Language translators may be helpful to overcome the communication barrier. Secondly, patient privacy is an issue that must be addressed; free-access communication media, although low-cost, are often not encrypted and are vulnerable to tapping or hacking by third parties. Thirdly, the affordability of treatment such as alteplase has to be considered; patients may be deprived of life-saving treatment if they reach a peripheral center within the window period but are not able to afford the costs of treatment [6]. Low cost alternatives such as tenecteplase has shown to be effective and can be a viable option [15,16]. In addition, government policies and local health insurance services should be planned and instituted to assure affordability of vital healthcare services.

Bearing in mind the potential downsides and the need for a comprehensive plan and infrastructure for telestroke services, this concept as a whole offers an economical, effective and expedient opportunity to improve access to stroke care in the South Asian region. The current COVID-19 pandemic has exacerbated the already poor access to stroke services in the rural areas of South Asia, which makes the search and installment of a timely solution all the more an imperative.

## Contributors

All authors contributed equally.

## Patient consent for publication

Not required.

## Data availability statement

No additional data are available.

## Provenance and peer review

Not commissioned, externally peer reviewed.

## Ethical approval

Not applicable.

## Sources of funding

None.

## Author contribution

Study concept and design: GN, JKY, and BPG. Drafting of the manuscript: JKY and GN. Manuscript revision and editing: GN, YKS and RRB. Supervision: BPG. All authors read and approved the final manuscript.

## Registration of research studies

Name of the registry: not applicable.

Unique Identifying number or registration ID: not applicable.

Hyperlink to your specific registration (must be publicly accessible and will be checked): not applicable.

## Guarantor

Dr. Gaurav Nepal and Dr. Jayant Kumar Yadav.

## Consent

Not applicable.

## Declaration of competing interest

None.
